# Fatal pulmonary hemorrhage in a horse during bronchoalveolar lavage – single case report

**DOI:** 10.1186/s12917-019-1922-9

**Published:** 2019-05-24

**Authors:** Mathilde S. Varegg, Kine M. Kløverød, Malin K. Austnes, Natalia Siwinska, Malwina Słowikowska, Agnieszka Zak, Janusz A. Madej, Malgorzata Kandefer-Gola, Rafal Ciaputa, Marcin Nowak, Artur Niedzwiedz

**Affiliations:** 1 Faculty of Veterinary Medicine, Wroclaw University of Environmental and Life Sciences, Wroclaw, Poland; 2 Department of Internal Diseases with Clinic for Horses, Dogs and Cats, Wroclaw University of Environmental and Life Sciences, Grunwaldzki 47, 50-366, Wroclaw, Poland; 3 Department of Pathology, Wroclaw University of Environmental and Life Sciences, Wroclaw, Poland

**Keywords:** BAL, Bronchoalveolar lavage, Horse, Samplings, Fatal hemorrhage

## Abstract

**Background:**

Pulmonary hemorrhage is a rare cause of death in horses. Hemorrhage within the respiratory tract has many causes, including mycosis of the guttural pouch, invasive procedures causing serious trauma to nasal conchae, or lung biopsy. We report on a rare case of a fatal pulmonary hemorrhage in a horse after a severe cough during bronchoalveolar lavage. To the best of our knowledge, this is the first report of spontaneous hemorrhage in a horse during bronchoalveolar lavage.

**Case presentation:**

A 21-year-old mare which belonged to the didactic herd of The Faculty of Veterinary Medicine underwent BAL procedure for training purposes. Clinical examination prior to the procedure did not reveal any abnormalities and the horse had been classified as healthy. The horse was sedated with 0.01 mg/kg of detomidine and 0.01 mg/kg of butorphanol. The silicon BAL catheter was passed through the nasal passage into the trachea and then into the bronchus. Before catheter was wedged, the mare began to cough heavily and massive haemorrhage from mouth and nostrils occurred. Despite fluid therapy, shock occurred within 15 min and the mare was euthanized. Upon necropsy, site of hemorrhage was identified in the left lobar caudal bronchi, from a large blood vessel running directly beneath the bronchial wall. Upon histology, a chronic lympho-plasmocytic inflammatory process in left bronchi was identified. Moreover, Masson’s trichrome staining revealed severe, perivascular fibrosis.

**Conclusion:**

Although BAL is a relatively safe procedure, and such complications should be treated as extremely rare, this case indicates that, in some individuals with specific subclinical problems, even mild physical force such as a cough can lead to rupture of the artery.

## Background

Severe hemorrhage from the respiratory tract in horses is mainly associated with mycosis of the guttural pouch, exercise-induced pulmonary hemorrhage (EIPH), invasive procedures causing serious trauma to nasal conchae, or lung biopsy [1, 2]. Other, rare conditions that can be a reason of respiratory bleeding in horses include aortobronchial fistula, aortic aneurysms or aortic rupture in young Friesian horses at the aortic arch or aortic root [3]. Moreover, fatal pulmonary hemorrhage associated with RTX toxin producing *Actinobacillus equuli* has been described in American Paint mare [4].

Bronchoalveolar lavage (BAL) in horses is a procedure used primarily for diagnosis of non-septic conditions, such as equine asthma or exercise-induced pulmonary hemorrhage (EIPH) [5]. During this procedure, cells and respiratory secretions are obtained from alveolar spaces for cytology.

The BAL can be performed either with an endoscope or blindly by equine BAL catheter. Horses should be sedated for both procedures. Performed blindly, the samples are most commonly taken from the right lung due to the position of the heart giving a straighter angle into the right bronchus [6]. Samples are taken from a localized part of the lung; therefore, BAL is only used in the diagnosis of diffuse lower airway disease. Overall, BAL is considered very safe and sufficiently sensitive to detect inflammation at the cytological level [7]. The most commonly reported BAL complications include a severe cough after a procedure, fever, and transient hypoxemia. These complications are relatively rare and spontaneously disappear within hours of the procedure [6, 7].

Fatal hemorrhage in a horse after severe cough during BAL procedure has not been reported before. The authors, therefore, report the first case of pulmonary hemorrhage followed by sudden death in a horse as a consequence of a severe cough during the BAL procedure.

## Case presentation

The 21-year-old mare belonged to the didactic herd of The Faculty of Veterinary Medicine, Wroclaw University of Environmental and Life Sciences. BAL was performed for teaching purposes. Clinical examination prior to the procedure did not reveal any abnormalities and the horse was classified as healthy. The horse was restrained in a stock and sedated with 0.01 mg/kg of detomidine (Domosedan, Orionpharma, Espoo, Finland) and 0.01 mg/kg of butorphanol (Morphasol, aniMedica, GmBH, Senden-Bösensell, Germany) [8]. A twitch was additionally used. A silicon BAL catheter (Large animal bronchoalveolar lavage catheter, Mila International Inc. Florence, KY, USA) was introduced to the ventral meatus through the left nostril, further passed in the trachea and bronchi. After the catheter was wedged in the bronchus, the cuff was inflated with 5 ml of air. The sudden movement of the mare made a reassessment of the catheter’s position necessary. The catheter was not sufficiently wedged, the cuff was deflated, and the catheter was introduced further. The mare began to cough heavily and massive hemorrhage from the nostrils and mouth followed. An attempt was made to place an endoscope (Karl Storz, 180 cm GmBH, Tuttlingen, Germany) into the airways to find the cause of hemorrhage, but due to profuse bleeding and coughing, this was impossible. A large gauged intravenous catheter was introduced, and the mare was given fluid therapy (Fresenius NaCl 0,9%, 3000 ml, Fresenius Kabi Polska Sp. z o.o., Warsaw, Poland). Despite fluid therapy, signs of hypovolemic shock occurred, and the mare collapsed 10 min after onset of bleeding. Within 5 min, due to the poor prognosis, the mare was euthanized with intravenous pentobarbital sodium injection (Morbital 133 mg/ml, 100 ml, Biowet Pulawy, Poland). The mare was sent to necropsy at the Department of Pathology, Faculty of Veterinary Medicine, Wroclaw University of Environmental and Life Sciences.

**Fig. 1:**
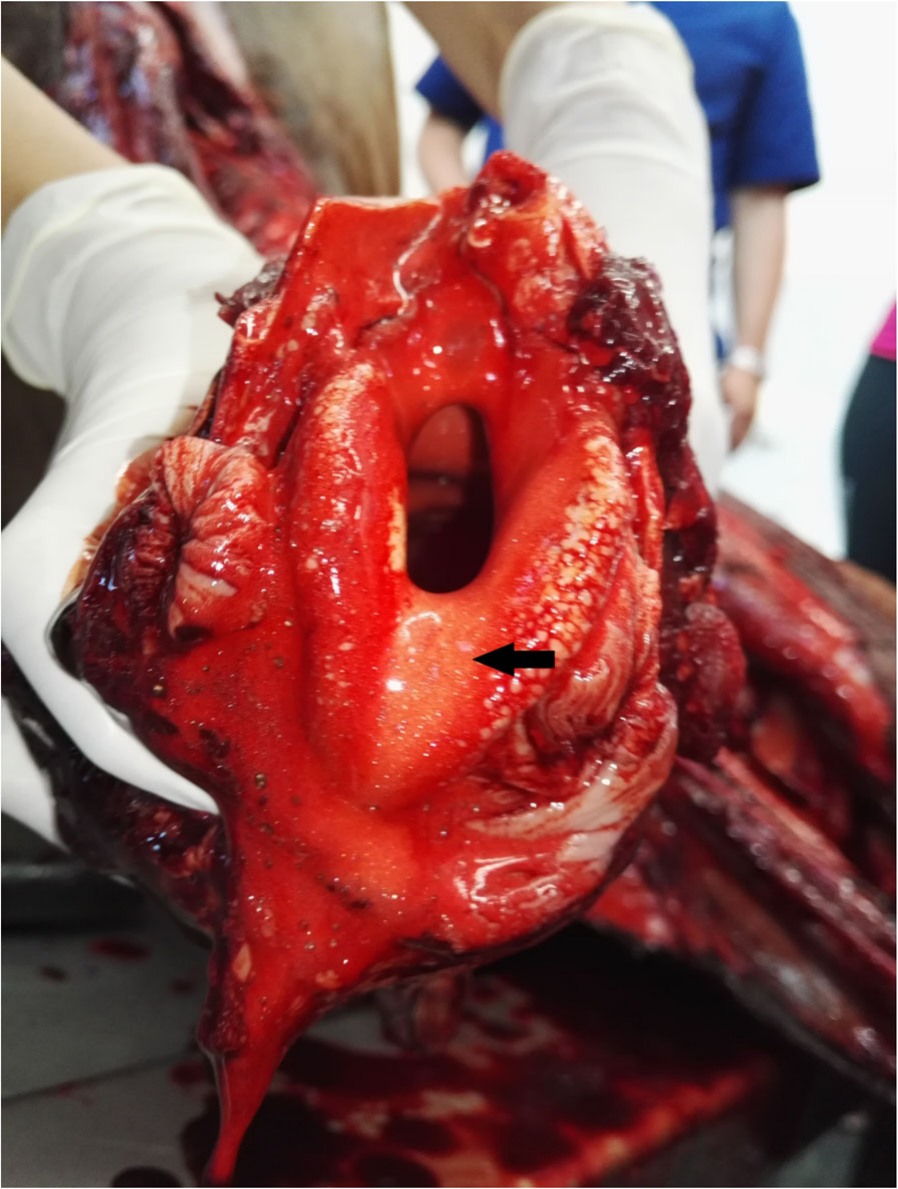
Cross section of trachea. The arrow shows dense, foamy, bloody fluid which filled the trachea lumen

**Fig. 2:**
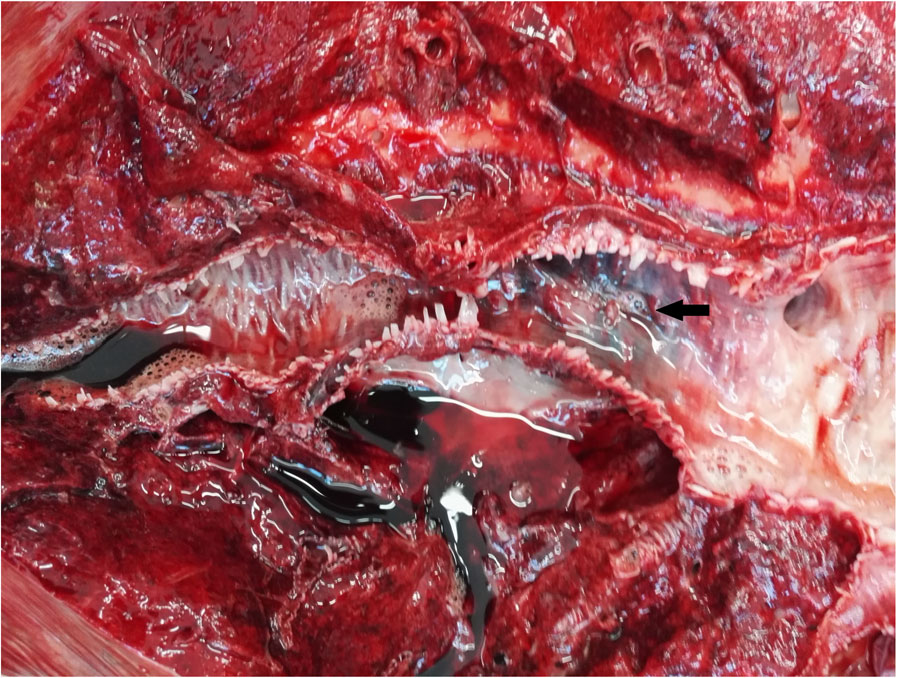
Cross section of bronchi and lungs. The arrow shows hemorrhagic focus visible on the bronchial wall. Inside the bronchi bloody fluid is visible

Horse necropsy was performed. The skin around nostrils and oral cavity were covered with blood. The nostrils, oral cavity, nasopharynx, pharynx, trachea and upper parts of the esophagus were filled with blood [Fig. 1]. Light, reddish, foamy, bloody fluid was observed in trachea which continued to the bifurcation and down to the large and small bronchioles, mainly in the left site. The left cranial lung lobe was hyperemic with several foci of hemorrhages [Fig. 2]. The cross-section of this lobe disclosed severe hemorrhagic areas mainly localized around bronchi. During macroscopic examination no sign of bronchial wall perforation was observed. The rest of the lung lobes were mildly hyperemic. The site of hemorrhage was identified in the left lobar caudal bronchi, from a large blood vessel running directly beneath the bronchial wall. The pericardial sac was filled with a small amount of serous fluid. The heart muscle and valves were not grossly abnormal, and the thoracic cavity did not reveal any other pathology.

**Fig. 3:**
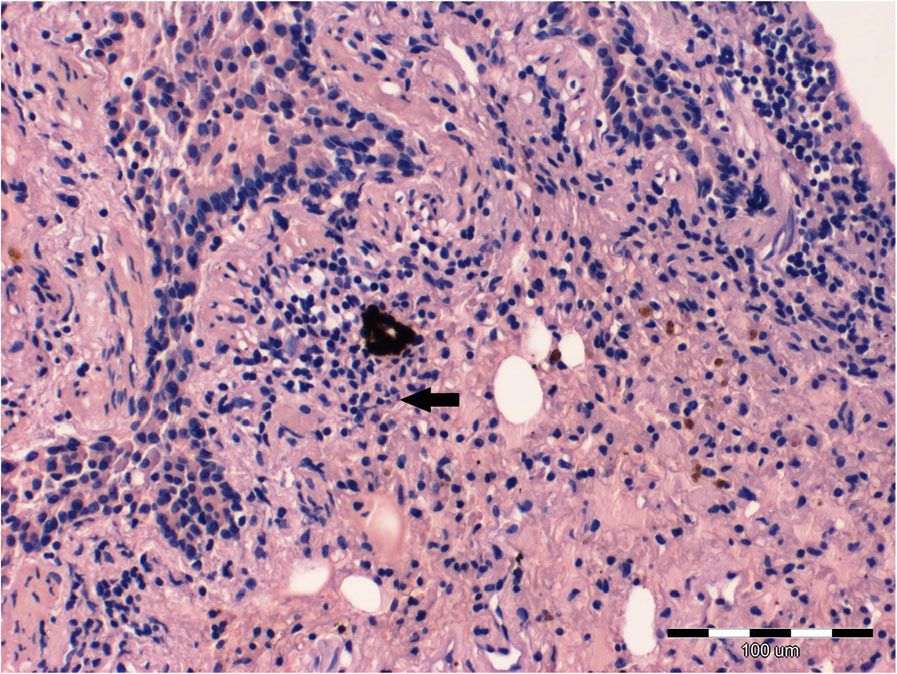
Lungs parenchyma. The arrow shows lympho-plasmocytic peribronchitits. Also light-pink, eosinophilic oedematic fluid is visible. H&E staining. 200x

**Fig. 4:**
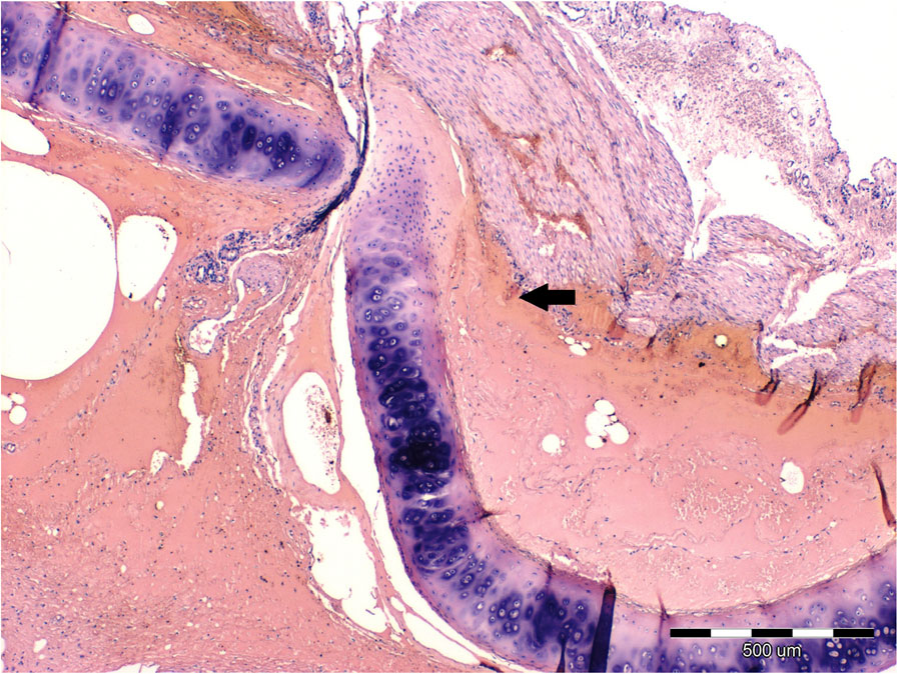
Lungs parenchyma. Massive, diffuse hemorrhage in the bronchial wall. The arrow shows accumulation of hemosiderin. H&E staining. 40x

**Fig. 5:**
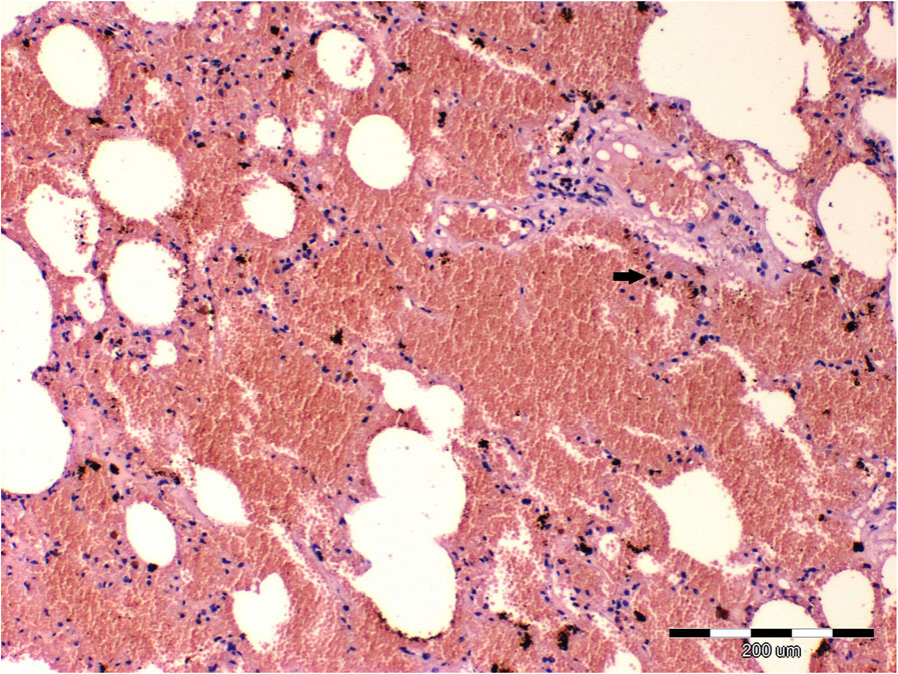
Lungs parenchyma. The arrow shows blood stasis. Accumulation of red blood cells and hemosiderin in the alveolar space are visible. H&E staining. 100x

**Fig. 6:**
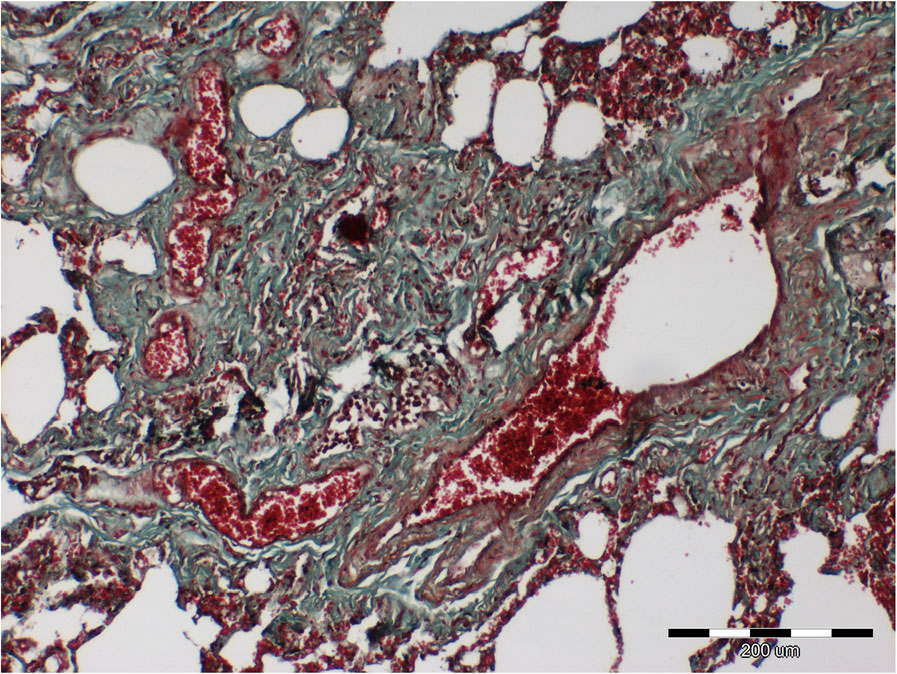
Lungs parenchyma. Severe perivascular fibrosis (green color) and lung hyperemia (red color). Masson-Goldner trichrome staining. 100x

**Fig. 7:**
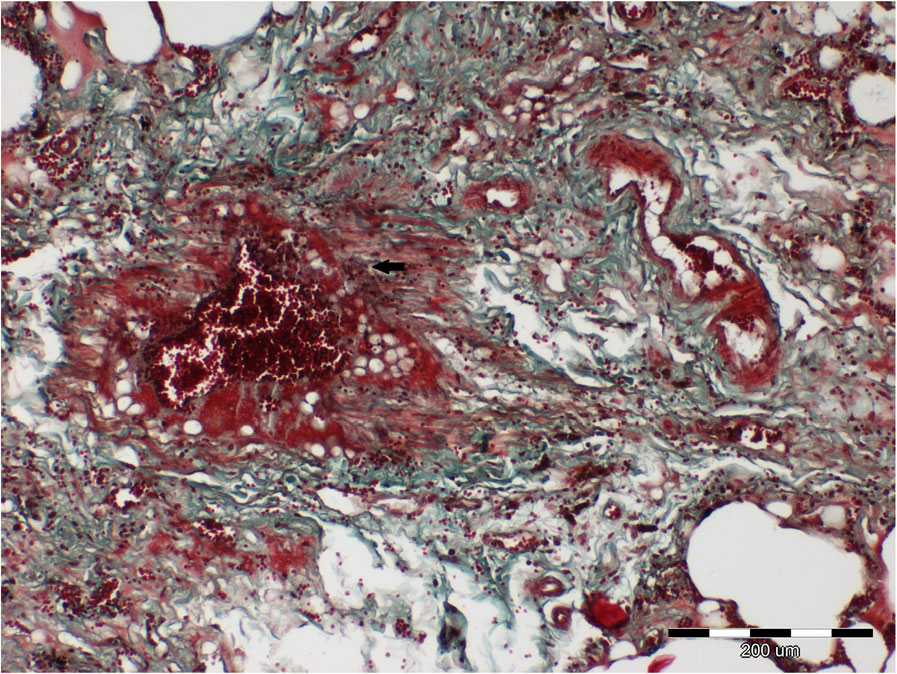
Lungs parenchyma. The arrow shows severe perivascular fibrosis which destroy blood vessel wall. Masson-Goldner trichrome staining. 100x

The histopathologic examination revealed a chronic, lympho-plasmocytic inflammation of the left bronchial wall [Fig. 3]. Severe, diffuse hemorrhage in alveoli was also observed [Fig. 4]. The accumulation of the red blood cells and subsequently hemosiderin in the alveolar space was disclosed [Fig. 5]. In addition, Masson’s trichrome staining was performed, which revealed severe, perivascular fibrosis [Figs. 6, 7].

The cause of death was hypovolemic shock and severe hypoxemia secondary to blood flooding alveoli, presumably from ruptured pulmonary or bronchial artery segment which was weakened as a consequence of severe perivascular fibrosis and chronic inflammatory process.

## Discussion and conclusions

Bronchoalveolar lavage is a relatively safe procedure and complications are uncommon and usually of minor importance and severity. Fatal hemorrhage during bronchoalveolar lavage has to the authors’ knowledge never been reported before and should be considered as extremely unusual.

A cough is initiated by irritant receptors in the airways. Stimulation of these receptors provokes a cough reflex. Although intratracheal administration of lidocaine proved to be the most reliable method to reduce the frequency and intensity of coughing in horses during BAL, we did not decide to use it. The main reason for it was economy and awareness that the procedure is carried out on a healthy animal, whose a cough is not severe due to e.g. airway inflammation. Moreover, we sedated the horse with IV administration of butorphanol, which also resulted in a significant reduction in the intensity of coughing [9].

In coughing horses, the thoraco-abdominal pressure rises > 100 cm H_2_O above ambient pressure [10]. In our patient, the cough reflex was likely overstimulated by the BAL catheter being in contact with the bronchial wall. The increase in pressure in this area during coughing might have been sufficient to rupture the inflamed vessel and the bronchial wall underneath. Spontaneous rupture of the artery in generally healthy patients with no definite trauma is extremely rare, and we found only a few cases in the existing literature, concerning human medicine. However, in those cases, some risk factors were observed. These risk factors included: elderly patients, females, patients on anticoagulation medication or having a cough [11–14]. This indicates that, in a generally healthy man, even mild physical force such as cough can cause injury of the artery. In our case, although considered as healthy, the histopathological examination revealed a chronic lympho-plasmocytic inflammatory process in the left bronchial wall, together with severe, perivascular fibrosis. This inflammatory process was located around a large pulmonary artery, which was likely a contributing factor to the rupture of the vessel. One report in a horse with fatal hemoptysis mentions small aggregates of lymphocytes, plasma cells, and macrophages in the tunica adventitia. Moreover, thoracic aorta showed medial necrosis and the mineralization and heterotopic bone formation [3]. Although there is some similarity with inflammatory infiltration, in our case we did not find any traces of mineralization. In man, the most common form of calcific vasculopathy is atherosclerosis, which is extremely rare in horses and has been reported in horses with *Dirofilaria* [15].

A healthy arterial wall has a strong muscular layer, and is unlikely to rupture, even under high pressure [16]. However, review of the human literature reveals numerous cases of spontaneous rupture of an intercostal artery, without any predisposing factors (ie. Ehlers danlos syndrome, Marfans disease, Neurofibromatosis Type I), or any associated illness or injury [17, 18].

Besides the above, human literature describe numerous cases of pulmonary/bronchial artery rupture. In vast amount, they refer to thoracic neoplasms, pulmonary tuberculosis, trauma, arteriosclerosis or complication of intravessel procedures [19]. Moreover, pulmonary and bronchial arteries thrombosis and aneurysms have been described in human, resulting in severe, life-threatening hemoptysis. This condition known as Hughes-Stovin syndrome manifests clinically through three phases: a first one involves symptoms of thrombophlebitis, a second one involves the formation of pulmonary artery aneurysms followed by the third phase of aneurysmal rupture which results in massive hemoptysis and death [20]. In horses, such syndrome hasn’t been described, and in our case exact site of artery rupture hasn’t been identified, thus, based on histology findings, we assume in our case different than in Hughes-Stovin syndrome pathophysiology.

Hypovolemic shock due to massive hemorrhage requires emergency procedures. According to literature, the therapy of acute blood loss depends on the form of hemorrhage [21]. The therapy of choice in uncontrollable pulmonary hemorrhage is hypotensive resuscitation. The goal is to maintain a minimal mean arterial blood pressure, to ensure an end-organ perfusion without potentiating blood loss. Conservative treatment with slow administration of whole blood, plasma, packed RBC (red blood cells) with plasma or isotonic crystalloids is indicated. Monitoring to ensure adequate minimal organ perfusion includes a mean arterial pressure of 60 mmHg, blood lactate concentration less than 4 mmol/L, blood pH greater than 7,25 and maintenance of urine production [22]. Due to the rapid progression of this case, monitoring of the patient’s parameters was impossible. As stated previously, within 10 min the mare collapsed due to hypovolemic shock despite therapy.

Pulmonary hemorrhage can be mild and transient, not requiring any intervention, as in the previously mentioned studies of lung biopsy complications. On the other hand, as in this case report, severe pulmonary hemorrhage resulted in hypovolemic shock and euthanasia due to the poor prognosis. Fatal pulmonary hemorrhage has not, to the authors’ knowledge, been reported as a complication of a severe cough during a standard bronchoalveolar lavage. Presumably, the cause of this severe complication was associated with an unidentified subclinical disease. Although BAL is a relatively safe procedure, and fatal hemorrhage should be treated as extremely unusual; this case indicates that, in some individuals with specific subclinical problems, even mild physical force such as a cough can lead to rupture of the artery.

## Data Availability

All data generated or analyzed during this study are included in this published article.

## Abbreviation

BAL: Bronchoalveolar lavage
